# A prospective observational within-person MRI morphometric study comparing ictal and interictal brain volumes in status migrainosus

**DOI:** 10.1016/j.ynirp.2026.100367

**Published:** 2026-06-11

**Authors:** Jennifer Robblee, Timothy Noah Hutson, Susan Criswell

**Affiliations:** Department of Neurology, Barrow Neurological Institute, St. Joseph's Hospital and Medical Center, Phoenix, AZ, USA

**Keywords:** Status migrainosus, MRI, Volumetric analysis, Migraine

## Abstract

**Objectives:**

To assess whether total masked brain volume differs during status migrainosus (SM) compared to post-SM interictal baseline using structural MRI.

**Methods:**

This prospective within-person study included 11 participants scanned during an SM attack and post-SM interictal baseline using T1-weighted MRI. FreeSurfer-based volumetric analysis was performed. Linear mixed effect models evaluated global and regional brain volume comparing SM and interictal baseline. The primary outcome (total brain volume) was adjusted for age and interictal pain severity; secondary grouped regional analyses (cerebral cortex, subcortical white matter, and subcortical gray matter) were adjusted for age.

**Results:**

Participants had a mean age of 50.1 (SD 17.6) years and 91% (10/11) were female. Median SM attack duration was 600 (IQR 264-936) hours. Total brain volume was higher during SM compared with interictal baseline (β = 8897.000 mm^3^, 95% CI 2478–15,317 mm^3^; p = 0.012). The cerebral cortex and deep gray matter both showed a non-significant decrease in volume. Subcortical white matter showed a non-significant increase in volume.

**Discussion:**

This within-person study demonstrates that total brain volume is higher during SM compared with the post-SM interictal state. The cause is unknown, but possible mechanisms include subclinical cytotoxic edema. Larger studies with more granular analyses are needed to clarify the mechanisms and clinical significance of these findings.

## Highlights

• Total brain volume is higher during status migrainosus compared to the interictal state.
• No significant ictal-interictal differences were found in cortex, white matter, or deep gray matter.
• Prospective study of a rare patient population, enabling ictal vs interictal within-subject comparison.

## Graphical abstract

**Figure:**
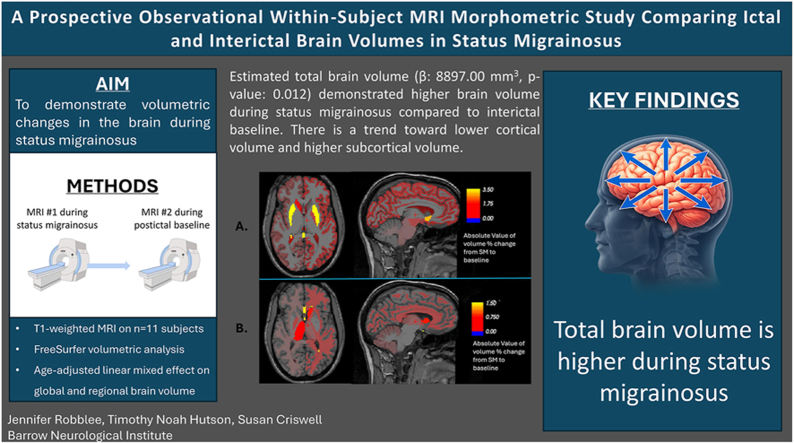

## 1 Introduction

Status migrainosus (SM) is a common but likely underdiagnosed migraine complication defined as a severe attack lasting greater than 72 h (Headache Classification Committee of the International Headache Society, 2018). Its epidemiology has not been well studied with reports ranging from 3% to almost 25% of patients with migraine with a prevalence of 43.9% among patients with migraine in subspecialty care(Andress-Rothrock et al., 2010; Beltramone and Donnet, 2014; Dorsey and Robblee, 2026; Orr et al., 2020; Pryse-Phillips et al., 2006). Attacks have a reported mean duration of 8.6 days to 4.8 weeks, but can last months with one retrospective cohort reporting a range up to 330 days (Beltramone and Donnet, 2014; Dorsey and Robblee, 2026; VanderPluym et al., 2023). This migraine complication causes severe disability and is associated with a higher risk of suicide (Harnod et al., 2018). The 1-year risk of SM recurrence is 14.8% (VanderPluym et al., 2023). Unfortunately, headache freedom following SM is only achieved in 20 to 25% of patients (Rozen, 2015). In contrast, in a typical migraine attack, approximately 40% of patients achieve pain freedom within 2 h, and 59% report meaningful pain relief at 2 h (Ferrari et al., 2002; Tfelt-Hansen and Diener, 2020). Despite the disability associated with this common migraine complication, little is known about its underlying pathophysiology.

The concept of SM evolved in the 1970s and 1980s, but few studies have examined its pathophysiology, with most existing reports focusing on secondary headache disorders presenting with an SM phenotype. One study identified abnormal peak plasma melatonin levels compared to the expected physiologic response in the setting of a melatonin infusion in 6 females with SM when compared to normal controls suggesting impaired pineal function. (Claustrat et al., 1997). Otherwise, there are case reports utilizing imaging during SM. One case report of MRI and ^18^F-fluorodeoxyglucose PET obtained ictally identified reversible changes consistent with vasogenic edema in the supratentorial white matter as well as the adjacent temporal and occipital cortices (Gentile et al., 2009). The second case report noted angiographic changes with areas of segmental stenosis contrasting with segmental dilatation, which the authors hypothesize related to neuroinflammation different than angiitis (Schulman and Hershey, 1991). A similar finding was reported in a case with dilatation of carotid and vertebrobasilar branches during a typical migraine attack (Masuzawa et al., 1983). Another case report noted a transient lesion in the splenium of the corpus callosum during SM (Samanta, 2015).

Outside of SM, multimodal neuroimaging studies in migraine have identified structural and functional differences across the disease spectrum using structural MRI, fMRI, MRI spectroscopy, EEG and magneto-physiological brain activity (MEG) (Gomez-Pilar et al., 2022). A systematic review and meta-analysis across 40 studies including 1616 patients and 1681 matched controls demonstrated multifocal gray matter changes in areas of the brain that process pain, sensation and emotion (Zhang et al., 2022). Findings across studies, however, have been inconsistent with reports of increased or decreased cortical thickness in various regions differing across MRI protocols and clinical context. Most of this work has focused on interictal imaging in episodic or chronic migraine, and interpretations typically center on cumulative disease burden or chronification.

In contrast, imaging during an active migraine attack is far more limited. One study by Amin et al. scanned 25 patients ictally and interictally with T1-weighted MRI (Amin et al., 2021). Of the 20 patients undergoing both scans, 15 patients were found to have reduced cortical thickness in the precentral, pericalcarine, and temporal cortices with increased hippocampal volume ictally. These findings suggest that structural measures can change rapidly and may reflect transient morphological or perfusion-related effects (Boes et al., 2018; Elvsåshagen et al., 2017; Gbyl et al., 2019). SM, a prolonged and disabling migraine complication, has been almost entirely excluded from research. Prior work in SM is limited to the above-described case reports, and no studies have used a within-person design to evaluate volumetric changes during an SM attack. Given the evidence that structural measures can shift acutely during migraine attacks, we hypothesized that SM may also be associated with acute volumetric changes. The goal of this study was to compare brain volumes during SM versus interictal baseline.

## 2 Methods

### 2.1 Overview

This study is a prospective within-person design that used structural MRI imaging in patients with SM to better understand in-vivo structural pathophysiology. Enrolled patients required a diagnosis of migraine, and during the ictal scan needed to meet the *International Criteria for Headache Disorders 3*rd *edition* (ICHD-3) diagnostic criteria for a SM attack with the additional requirement of having tried acute treatment within the initial 24 h of the attack. Patients were imaged ictally and interictally using T1-weighted images. At the time of consent, patient age, sex, migraine diagnosis per ICHD-3, current preventive and rescue medications, disease duration, presence of aura, Migraine Disability Score (MIDAS) (Stewart et al., 2000), monthly migraine days, monthly headache days, average migraine severity, and history of prior SM were recorded. During the SM attack, participants reported the duration of the attack, occurrence with aura, attack severity, pain lateralization, and time of last acute medication. The primary outcome was ictal total brain volume during SM compared to the interictal baseline.

### 2.2 Participants

Patients with episodic or chronic migraine were recruited from a specialty headache clinic over a one-year period.

The inclusion criteria were patients aged 18 years and above, established diagnosis of migraine per the ICHD-3 criteria, and provision of written consent. Acutely, the patient had to be mid-SM attack that met SM ICHD-3 criteria, and they had to receive an evidence-based acute treatment within 24 h of attack onset (including triptans, gepants, ditans, dihydroergotamine, neuromodulation devices, anti-dopaminergics, and non-steroidal anti-inflammatories as described in Ailani et al.) to exclude under-treated attacks (Ailani et al., 2021). The exclusion criteria were inability to provide reliable clinical history regarding SM attack details, other primary or secondary causes of headache, pregnancy or breastfeeding, inability to undergo an MRI scan, and history of neurological disease at the investigator's discretion. Patients with chronic migraine were included if they had a distinct SM attack, but participants with monitored interictal headache pain intensity (≥5/10) were excluded to ensure that the ictal and interictal phases could be distinguished. Additionally, their recorded interictal pain severity had to be at least 3 points below the recorded peak SM severity for inclusion in analysis. Participants were asked to minimize or avoid acute treatment within 6 h of the ictal MRI to reduce potential confounding from recent medication use. However, because SM is a severe and disabling attack lasting days to weeks, strict adherence to this requirement was felt to be unreasonable. Therefore, use of acute medications within 6 h was not treated as a formal exclusion criterion. The time since last acute treatment was recorded for each participant and is reported in the Results.

### 2.3 Protocol

Each participant underwent one ictal MRI during SM and one interictal baseline MRI; no repeated MRI measurements were acquired within either state. The first ictal scan was performed during an acute episode of SM, which required imaging to occur after 72 h of headache duration to meet ICHD-3 criteria but before the end of the headache phase. The second scan was done interictally when no migraine attack or SM was occurring. Mild baseline headache not meeting ICHD-3 migraine criteria (pain severity <5/10) during the inter-ictal scan was allowed.

Images were obtained from a research-dedicated 3-T Philips Ingenia MRI scanner. A standard sagittal T1-weighted anatomical image was acquired using a 3D magnetization-prepared rapid acquisition gradient echo (MPRAGE) sequence with the following acquisition parameters: relaxation time/echo time (TR/TE), 6.9/3.164 ms; acquisition matrix, 256 × 256; voxel size, 1 × 1 × 1 mm^3^; slice thickness, 1 mm; flip angle = 90, field of view (FOV) was square 241 mm wide. All MRIs were reviewed by a neuroradiologist prior to processing.

### 2.4 MRI processing

We used FreeSurfer to perform brain extraction from T1-weighted MPRAGE. FreeSurfer volumetric outputs were derived in native space. The final output was the volume of all segmented brain regions. Absolute brain volumes (mm^3^) were analyzed using morphometric analysis from FreeSurfer v6.0 (Martinos Center for Biomedical Imaging, Boston, MA) (Laboratory for Computational Neuroimaging, Athinoula A. Martinos Center, 2018).

Total brain volume was defined as masked volume of brain, brainstem, and ventricles. Total ventricular volume, included to contextualize change in brain volume, was calculated as the sum of the FreeSurfer volumes for the left and right lateral ventricles, left and right inferior lateral ventricles, third ventricle, and fourth ventricle.

### 2.5 Primary outcome

The primary outcome was total brain volume compared between the SM MRI and the interictal baseline MRI across participants. Age was included as an a priori covariate given known effects on brain volume. Post-SM pain severity (0–10) was also evaluated as a covariate, given the possibility that residual pain intensity at the time of the interictal scan could influence volumetric measures. Sensitivity analysis was performed with sex as a covariate, although interpretation was limited by the inclusion of only one male participant.

### 2.6 Secondary outcomes

We explored the change in volume between SM and baseline in three major regions of interest (ROIs) as the secondary outcome: the cerebral cortex, the subcortical white matter, and the deep gray matter to capture the major supratentorial subregions. The models were adjusted for age. Absolute volume (mm^3^) for each region was approximated using FreeSurfer morphometry. The cerebral cortex and subcortical white matter are standard output regions for FreeSurfer. The deep gray matter ROI was calculated as the sum of the bilateral caudate, putamen, pallidum, thalamus, and accumbens volumes.

### 2.7 Statistical analysis

To assess the clinical data, we used both descriptive and inferential statistics. Descriptive statistics included means and standard deviation (SD) for continuous variables. When the data did not meet the assumptions of a normal distribution, we used nonparametric summaries (medians and interquartile ranges). Each variable of interest was evaluated via Shapiro-Wilk test for normality. There were no missing data.

A linear mixed effect model (LME) was employed to evaluate the change in total brain volume with brain state, attack (SM) versus interictal baseline. The model was adjusted for age and post-SM interictal pain severity. As a sensitivity analysis, we also adjusted for sex in a separate model. The models were designed so that a positive β coefficient estimate for volume would correspond to a higher volume during SM with α = 0.05 and we calculated effect sizes with 95% confidence intervals (95% CI).

We employed LME to assess the association between brain state and our 3 secondary ROI volumes with adjustment for age. As for the primary analysis, the models were designed so that a positive β coefficient estimate for volume would correspond to a higher volume during SM. False discovery rate (FDR) was used to account for the multiple analyses across the three ROIs. Post hoc, we explored the correlation between percent change of total brain volume and the following clinical measures: monthly migraine days, monthly headache days, attack severity, disease duration, and SM attack duration at the time of ictal MRI. As an additional sensitivity analysis, we explored the correlation between percent change in total masked brain volume and the time between the ictal and post-SM interictal MRI.

MATLAB 2024B (Statistics and Machine Learning Toolbox, MathWorks, Natick, MA) and R version 4.4.2 (R Foundation for Statistical Computing, Vienna, Austria) were used in estimation of descriptive statistics and in statistical tests (R Core Team, 2024; The MathWorks Inc., 2024).

### 2.8 Bias mitigation statement

This was a prospective within-subject observational study and did not include randomization or blinding. To reduce selection bias, participants were enrolled using prespecified inclusion and exclusion criteria. To reduce measurement bias, ictal and interictal MRI scans were acquired on the same 3T scanner using the same acquisition parameters. To reduce confounding from between-person anatomical variability, the primary analyses used within-subject comparisons between ictal and interictal scans. Relevant clinical factors, including SM attack duration, attack severity, baseline headache severity, and time from last acute medication use to ictal MRI, were recorded to contextualize potential sources of bias.

### 2.9 Ethics approval and consent to participate

Written informed consent was obtained by all participants. This study was approved by the St Joseph's Hospital and Health Center IRB (23-500-389-30-10).

## 3 Results

### 3.1 Sample characteristics

The study enrolled 11 participants, who completed both ictal and interictal MRIs, with a mean age of 50.1 years; however, the range was wide (range 29-77 years; SD of 17.7 years). The majority were women (91% (10/11)) and predominantly self-reported to be white. All but one participant had a baseline diagnosis of chronic migraine, though many had converted to episodic migraine on current treatment. At baseline, only 3 participants had a diagnosis of migraine with aura. Monthly migraine days were 7.8 ± 5.6 days with an average migraine attack severity of 8.0 ± 1.6 out of 10. Monthly headache days were self-reported as 20.0 (6.0, 30.0) with interictal severity reported as 2.0 (Q1 0.0, Q3 3.0). All patients reported at least one previous SM attack. See Table 1 for a summary. See Supplementary Table 1 for an overview of the data for all 11 participants.

**Table 1:** Participant characteristics (n = 11).

Age (Years, Mean (SD))	50.1 (17.6)
Female gender (n(%))	10 (91%)
Race (n(%))	
White	9 (82%)
Black	1 (9%)
Multiple	1 (9%)
Diagnosis at Baseline (n (%))	
Episodic migraine	1 (9%)
Chronic migraine^a^	10 (91%)
With aura	3 (27%)
Without aura	8 (73%)
Migraine characteristics	
Monthly headache days (Median (IQR))	20.0 (6.0, 30.0)
Monthly migraine days (Mean (SD))	7.8 (5.6)
MIDAS (Mean (SD))	160.6 (51.9)
Typical attack severity (Mean (SD))	8.0 (1.6)
Typical attack duration (Hours, Median (IQR))	5.0 (3.0, 24.0)
Disease duration (Years, Mean (SD))	20.8 (11.1)
Prior status migrainosus (n(%))	11 (100%)
Preventive treatment (n(%))	
OnabotulinumtoxinA	7 (64%)
CGRP monoclonal antibody	3 (27%)
Gepant	1 (9%)
Topiramate	0 (0%)
Divalproex/valproate	1 (9%)
Beta Blocker	1 (9%)
Candesartan or lisinopril	0 (0%)
Amitriptyline	1 (9%)
Venlafaxine	1 (9%)
Memantine	1 (9%)
Nerve Blocks	3 (27%)
Other	7 (64%)

SD = Standard deviation, IQR = interquartile range, MIDAS = Migraine Disability Assessment, CGRP = calcitonin gene-related peptide.

a Includes patients with chronic migraine converted to episodic migraine on treatment.

The SM attack duration was recorded at the time of the ictal MRI and again after the attack. The total SM attack duration was right-skewed (w = 0.778, p-value = 0.005) with a median of several days (600 h) duration but could last for months (interquartile range of 264 to 936 h with a minimum of 9 days and maximum of 112 days). The time between the ictal and interictal MRI was a median of 8.8 days (Q1 6.0, Q3 15.5) with a range of 1.9 to 38.7 days. See Table 2. During the attack, 5 participants self-reported a visual aura despite 2 having no history of aura. Similarly, 7 participants self-reported a non-visual aura despite only one of them having a previous aura history. Participants were asked to refrain from using acute treatments within 6 h of the MRI when possible, but this was not a formal exclusion criterion given the severity of SM. The average time between last acute medication use and the ictal MRI was 8.6 h (SD 7.3), and this distribution was approximately normal.

**Table 2:** Status migrainosus attack characteristics (n = 11).

Attack duration (Hours, Median (IQR))	
At time of ictal MRI	191 (135, 372)
Total duration	600 (264, 936)
Peak attack severity (Mean (SD))	8.6 (0.9)
Pain laterality (n(%))	
Right	3 (27%)
Left	2 (18%)
Bilateral	6 (55%)
Associated aura (n(%))	
Visual aura	5 (46%)
Non-visual aura	7 (63%)
Acute treatment during status migrainosus (n(%))	
Triptan	3 (27%)
Gepant	2 (18%)
Ditan	1 (9%)
NSAIDs	4 (36%)
Acetaminophen	2 (18%)
Steroids	1 (9%)
Neuroleptic	1 (9%)
Parenteral treatment	0 (0%)
Other	1 (9%)

IQR = interquartile range, SD = standard deviation, NSAIDs = non-steroidal anti-inflammatory drugs.

### 3.2 Comparisons in total brain volume between SM and interictal baseline

In the primary outcome model adjusted for age and interictal pain severity, ictal brain volume was higher compared to interictal baseline (β = 8897.00 mm^3^, 95% CI 2478-15317 mm^3^; p = 0.012). This increase corresponds to a large standardized effect size of 1.32 standard deviation units (95% CI 0.37-2.27).

Interictal pain severity was not significantly associated with total brain volume (β = 8989 mm^3^, 95% CI –52,512 to 70,489 mm^3^, p = 0.745), and likelihood ratio testing indicated that its inclusion did not improve model fit (p = 0.694). Importantly, the ictal–interictal effect remained unchanged in the age-adjusted model without post-status pain. Sensitivity analysis using the 10 female participants, with the single male excluded, did not change the ictal–interictal effect indicating that sex itself was not significantly associated with total brain volume.

**Fig. 1:**
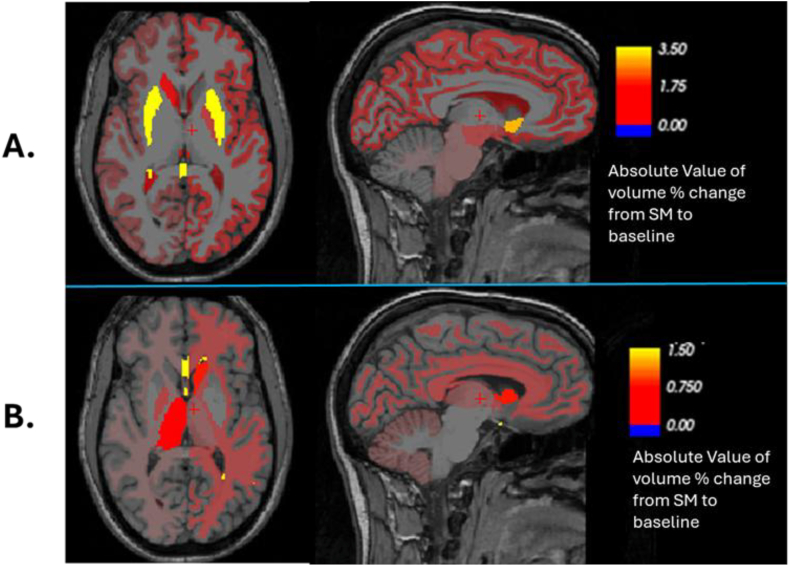
Absolute value of the percent volume change during status migrainosus (SM) compared with the interictal baseline. **Panel A** (top row) displays regions of decreased ictal volume, visualized on a 0.00 to 3.50 percent scale. **Panel B** (bottom row) displays regions of increased ictal volume, visualized on a 0.00 to 1.50 percent scale. Separate scales are used to preserve the dynamic range within each panel, as the magnitude of volume decreases substantially exceeds that of increases. Axial and sagittal slices in each panel show the spatial distribution and relative magnitude of these changes.

To better visualize brain-wide changes in volume, we pseudo-colored percent change in volume from SM to baseline in brain regions superimposed onto an anonymized MRI in Fig. 1. Regions within this figure were not constrained to our generalized groupings for visualization purposes. Notable in these figures is that while global brain volume is higher during SM, the general regional direction is for lower cortical volume and higher subcortical volume.

### 3.3 Comparisons in grouped ROI volumes between SM and interictal baseline

**Fig. 2:**
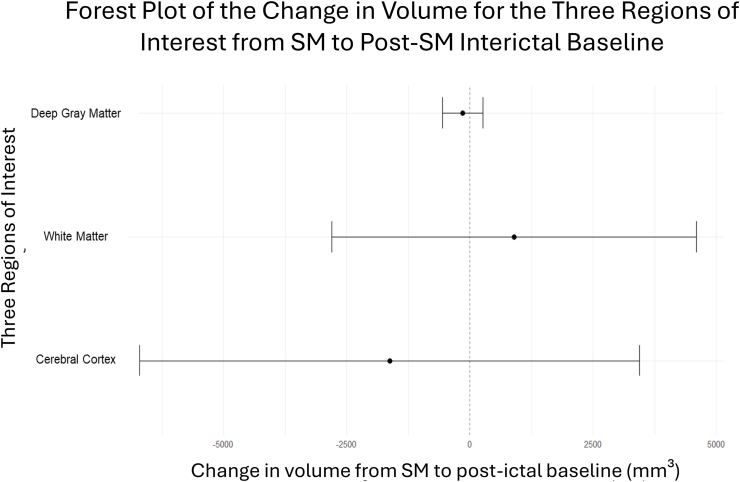
Forest plot for the three regions of interest (ROIs) with change in volume on the x-axis and the ROIs on the y-axis. There was a non-significant pattern toward lower volume for cortical and deep gray matter and higher volume for the white matter.

None of the three grouped ROIs demonstrated statistically significant differences between SM and interictal baseline scans. The cerebral cortex showed a non-significant decrease in volume (β = −1629.24 mm^3^, 95% CI –6702 to 3444 mm^3^; p = 0.491, FDR-adjusted p = 0.599; standardized effect size d = −0.03, 95% CI –0.14 to 0.07). Subcortical white matter showed a non-significant increase (β = 901.36 mm^3^, 95% CI –2800.75 to 4603.47 mm^3^; p = 0.599, FDR-adjusted p = 0.599; d = 0.020, 95% CI –0.070 to 0.110). The deep gray matter showed a non-significant decrease (β = −147.56 mm^3^, 95% CI –560.01 to 264.88 mm^3^; p = 0.444, FDR-adjusted p = 0.599; d = −0.040, 95% CI –0.130 to 0.060). Standardized effect sizes were close to zero across all three grouped ROIs, indicating minimal global differences between ictal and interictal states at this level of analysis. See Fig. 2 for forest plot visualization.

Ventricular volume was also reviewed to contextualize change in total brain volume. Ventricular compartment analyses did not show evidence of increased ventricular volume. There were no significant associations for the average ventricle volumes (β = 22.33 mm^3^, 95% CI -530.31 to 574.96 mm^3^, p = 0.930). Individual ventricle analyses were also non-significant, with the exception of the left inferior lateral ventricle, which showed a nominally smaller volume during SM (β = −40.364 mm^3^, 95% CI -73.535 to −7.192 mm^3^, p = 0.038), a finding in the opposite direction from what would be expected if ventricular enlargement were driving total brain volume increases.

### 3.4 Clinical associations with SM changes in volume

Post hoc Spearman correlations showed no significant association between percent change in total masked brain volume and monthly migraine days (ρ = 0.16, p = 0.63), monthly headache days (ρ = 0.24, p = 0.48), SM peak severity (ρ = 0.06, p = 0.87), typical migraine attack severity (ρ = −0.12, p = 0.73), disease duration (ρ = 0.22, p = 0.52), SM duration at the time of ictal MRI (ρ = 0.23, p = 0.50). As a sensitivity analysis, we tested whether the within-person change in masked brain volume was associated with the number of days between the ictal SM MRI and the subsequent post-SM interictal MRI. There was no statistically significant association between scan interval and change in masked brain volume using Spearman correlation (ρ = 0.35, p = 0.298). A supportive simple linear regression showed a similar nonsignificant association (β = 588.3 mm^3^/day, p = 0.104).

## 4 Discussion

The primary outcome using volumetric analysis demonstrated an increase in total brain volume during SM compared to the post-SM interictal state. Adjustment for age, sex, and interictal pain severity did not alter the observed ictal–interictal difference, supporting that these factors did not confound the primary effect. As this analysis is a within-person comparison on the same MRI machine, the observed change is more likely to represent a true biological change. Because pre-attack MRI was not acquired, the post-attack scan can serve only as an interictal reference point, not a proven pre-ictal baseline; nevertheless, phase-resolved migraine MRI studies show that some structural measures can vary across the migraine cycle and may return toward interictal values after the attack (Amin et al., 2021; Fouto et al., 2025). Prior continuous-scanning work demonstrates that migraine is a cyclic disorder with phase-locked brain changes, and that post-ictal functional measures return toward interictal patterns (Schulte and May, 2016; Schulte et al., 2020). A sensitivity analysis of our data evaluating whether the within-person change in masked brain volume was associated with the number of days between the ictal SM MRI and the subsequent post-SM interictal MRI showed no correlation. This finding does not support scan interval as the primary explanation for the observed within-person difference in masked brain volume, although the small sample size limits inference. Thus, the post-ictal scan can reasonably approximate an interictal reference state, although residual post-ictal effects cannot be excluded.

In contrast, the three grouped ROIs (cerebral cortex, subcortical white matter, and deep gray matter) did not show statistically significant differences. The direction of effects suggested lower cortical and deep gray matter volume and higher subcortical white matter volume ictally, but the standardized effect sizes were close to zero, indicating that global volumetric changes in these large regions are minimal. This pattern suggests that SM ictal changes may be multifocal rather than broadly distributed, consistent with a prior imaging study that found attack-related reductions cortical thickness restricted to the precentral, pericalcarine, and temporal cortices during a standard migraine attack (Amin et al., 2021). In our study, there may be localized changes in specific cortical and subcortical regions, but these occur across different tissue categories and are therefore obscured when volumes are grouped into broad regional bins. A larger sample size will be needed to better assess granular, region-specific changes.

### 4.1 Potential mechanisms for global brain volume increase

This study was not designed to determine mechanisms underlying the higher brain volume observed during SM. One speculative possibility is subtle, transient cytotoxic edema related to prolonged ictal physiology, given prior reports of increased glutamate during migraine attacks and biologic plausibility for glutamate-mediated excitotoxic swelling when clearance is impaired (Benbow and Cairns, 2021; Ferrari et al., 1990; Karsan et al., 2025; Neves et al., 2023; Peres et al., 2004; Ramadan, 2003; Ribeiro et al., 2024; Tehse and Taghibiglou, 2019; Yang et al., 2024a, 2024b; Zhou et al., 2020). An alternative, also speculative possibility is altered intracranial pressure or venous outflow physiology in a subset of patients with SM or refractory migraine (Abou-Mrad and Alaraj, 2024; Bono et al., 2010; De Simone et al., 2011, 2014, 2017; Favoni et al., 2019; Fofi et al., 2012; Giani et al., 2019; Schartz et al., 2025, 2026). Ventricular compartment analyses did not show evidence of ventricular enlargement accompanying the total brain volume finding. These hypotheses are not tested here and require dedicated multimodal studies.

### 4.2 Effect of attack duration

SM is a severe attack lasting more than 72 h, but can continue for weeks with no maximum duration including in the ICHD-3 diagnostic criteria (Headache Classification Committee of the International Headache Society, 2018). In our SM cohort, some participants had a SM attack that lasted months before resolving. How the total duration of a migraine or SM attack, especially long attacks like those seen in this study, affects structural MRI results is unknown as our study was not designed to capture changes longitudinally over the course of SM. There are few studies that have described the typical SM duration with study results ranging from 4 days to months (Akhtar et al., 2001; Beltramone and Donnet, 2014; Dorsey and Robblee, 2026; VanderPluym et al., 2023). We did not find an association between attack duration and global brain volumes in this small cohort, but further studies designed to assess longitudinal changes over the course of a SM attack are required to better assess any possible relationship.

### 4.3 Limitations

This study has several limitations. It was designed as a pilot, within-subject MRI volumetry study (n = 11; 2 scans per participant). In this context, our within-subject design, repeated measures, and statistically significant volumetric findings support the validity of the observed effect and provide preliminary effect size estimates; however, we acknowledge that the precision of these estimates and their generalizability are limited and will require confirmation in larger, prospectively powered cohorts. Although our sample size was small, similar sample sizes are common in MRI studies, with a recent analysis reporting that 96% of highly cited functional MRI studies have a median sample size of 12 (Szucs and Ioannidis, 2019). Statistically significant findings have been reported in studies with comparable or only modestly larger numbers of participants (Amin et al., 2021; Bilgiç et al., 2016; Yiğit et al., 2025). As a pilot study, we believe the findings of this work can serve as a steppingstone for larger studies investigating biomarkers and mechanisms of SM.

There was no blurring, ghosting artifacts, and a loss of sharp, detailed edges from head movement, and all participants were able to complete the MRI scans despite being in severe pain during the ictal scans. Recent volumetric MRI studies in other neurologic conditions demonstrate that quantitative volumetry can detect disease-associated regional brain differences, although findings vary by pathology, chronicity, comparator group, and segmentation approach (Gokoglu et al., 2026; Gökoğlu et al., 2025). Our study differs by focusing on acute within-person state-related volumetric change during SM rather than chronic between-group structural differences. Prior comparison studies report generally good-to-excellent agreement between FreeSurfer and other automated segmentation tools across many brain regions (Gokoglu et al., 2026; Gökoğlu et al., 2025), though cross-platform replication may further strengthen generalizability.

Another limitation is that the study included one ictal MRI and one subsequent post-SM interictal MRI, without a pre-ictal MRI. Given the unpredictable nature of SM, the feasibility of capturing a pre-ictal baseline scan is low. While the post-SM interictal scan cannot be assumed to represent the participant's pre-attack baseline, there is precedent in the literature for using the post-ictal scan to represent a patient's baseline (Amin et al., 2021), and prior longitudinal neuroimaging studies have shown that the post-ictal scan approximates the pre-ictal scan (Schulte and May, 2016; Schulte et al., 2020). We therefore interpret the primary finding as a within-person difference between ictal SM and the subsequent post-SM interictal state, but do not claim recovery to pre-ictal baseline, though this hypothesis is biologically plausible.

Study demographics like sex, race, and ethnicity should also be considered. Menstrual cycle phase was not recorded at the time of MRI acquisition. Given that 10 of 11 participants were female, menstrual phase represents a plausible unmeasured time-dependent source of variability in volumetric measures (Hagemann et al., 2011). The paired within-subject design reduces, but does not eliminate, this concern. However, studies have shown that any volumetric change related to menstrual cycle is modest, and multiple studies have shown regional effects without total brain volume effects (Meeker et al., 2020; Pletzer et al., 2018; Rizor et al., 2024). Future studies should prospectively record cycle timing, hormonal contraceptive use, and, when feasible, hormone levels at each scan. Race was reported as a descriptive sample characteristic only as the small sample size precluded meaningful assessment of racial differences. In a within-subject design, not controlling for race/ethnicity is less critical for internal validity, but it still limits external validity and interpretability. Notably, Liu et al. (2025) found that intracranial-volume normalization largely attenuated race-related volumetric differences in their cohort (Liu et al., 2025).

The heterogenous use of medications including acute medications during SM may have influenced the results, however participants were asked to avoid medications in the 6 h prior to the ictal MRI to limit their effect. There was a mean of 8.6 h between last acute medication use and the ictal MRI. Pain laterality during the SM attack was also heterogenous, which could influence regional averages.

Several modeling assumptions also warrant caution. The LME model included the interictal pain severity, which was not found to be a confounder, but the assumption of a linear relationship between age and volumetric change may not hold across the wide age range in this sample. Grouped ROI analyses were used to reduce multiple comparisons, but this approach may mask effects at the level of anatomical regions. Effect sizes and 95% CI were reported to improve interpretability, though they remain imprecise in a small sample. FDR correction was applied to reduce false positives, which further limited the ability to detect regional differences.

Finally, only a single ictal MRI followed by a post-ictal baseline MRI was obtained. There is no pre-ictal baseline MRI to ensure pre- and post-SM volumes were the same, which would require longitudinal studies of SM patients. SM was also not imaged at multiple time points to assess ictal changes over the course of an SM attack. However, this is a highly innovative SM study capturing ictal changes in a very challenging cohort. Further, this is a within-person comparison on the same MRI machine, supporting that observed differences reflect true biological change rather than artifact.

### 4.4 Future directions

Although preliminary, these findings highlight the potential of volumetric MRI as a biomarker for SM. Future studies with larger sample sizes will be needed to incorporate more granular regional analyses to determine whether specific cortical or subcortical structures drive the observed global change. Longitudinal imaging across the course of SM attacks will also be important to characterize temporal dynamics in both global and regional volumes. Finally, integration with functional and diffusion imaging may help link localized structural changes to underlying pathophysiological mechanisms and clinical outcomes.

## 5 Conclusion

This within-person study found that total brain volume is higher during status migrainosus compared with the post-status migrainosus interictal state. Larger studies with more granular regional analyses and longitudinal imaging are needed to clarify the mechanisms and clinical significance of these findings.

## Ethics approval and consent to participate

Written consent was received by all participants. This study was approved by the St Joseph's Hospital and Health Center Institutional Review Board (23-500-389-30-10).

## Preprint server

None.

## AI statement

Artificial intelligence tools (ChatGPT, OpenAI) were used solely to assist with language editing, grammar, readability, and troubleshooting minor coding errors during data analysis. These tools were not used for data generation, data analysis, interpretation of results, figure generation, or scientific decision-making. All analyses, results, interpretations, and manuscript content were reviewed and verified. The authors take full responsibility.

## Funding

Funding was received through a 10.13039/100009797Barrow Neurological Foundation grant.

## CRediT authorship contribution statement

**Jennifer Robblee:** Conceptualization, Data curation, Formal analysis, Funding acquisition, Investigation, Methodology, Project administration, Visualization, Writing – original draft, Writing – review & editing. **Timothy Noah Hutson:** Data curation, Formal analysis, Investigation, Methodology, Validation, Visualization, Writing – review & editing. **Susan Criswell:** Conceptualization, Data curation, Funding acquisition, Investigation, Methodology, Supervision, Validation, Writing – review & editing.

## Declaration of competing interest

Dr. Robblee discloses grant support from Barrow Neurological Foundation, investigator support from Eli Lilly and Abbvie, as well as paid editorial relationships with MedLink Neurology and Neurodiem. Dr. Robblee has received personal compensation for serving on advisory boards for Allergan and Abbvie, and Tonix. Dr Robblee also discloses that a family member has partial ownership of Scottsdale Providence Recovery Center.

Dr. Hutson discloses prior grant support from Barrow Neurological Foundation, NIH STTR 1R41NS132627-01, and NIHR01NS129643.

Dr. Criswell receives research support from the following government and nongovernmental organizations: NIEHS R01ES029524, R01OH011661, R01ES021488, and the Barrow Neurological Foundation.

## Data Availability

Data will be made available on request.
