# Supplementary material for: A prospective observational within-person MRI morphometric study comparing ictal and interictal brain volumes in status migrainosus
Source: Neuroimage Rep. 2026 Jun 11;6(3):100367. doi: 10.1016/j.ynirp.2026.100367 (PMC13276324; doi:10.1016/j.ynirp.2026.100367)
Supplement: Multimedia component 1 [file mmc1.docx]

Supplementary Material 1

| **Supplementary Table 1. Complete data for each participant** | | | | | | | | | | | |
| --- | --- | --- | --- | --- | --- | --- | --- | --- | --- | --- | --- |
|  | **#1** | **#2** | **#3** | **#4** | **#5** | **#6** | **#7** | **#8** | **#9** | **#10** | **#11** |
| **BASELINE MIGRAINE CHARACTERISTICS** | | | | | | | | | | | |
| **Aura** | N | Y | N | N | N | N | N | N | Y | N | Y |
| **MHD** | 3 | 30 | 6 | 30 | 30 | 25 | 6 | 12 | 20 | 30 | 29 |
| **MMD** | 1 | 12 | 2^ | 15 | 3 | 3 | 6 | 7 | 15 | 12 | 15 |
| **Attack**  **Severity (x/10)** | 8 | 10 | 10 | 8 | 9 | 7 | 5 | 4 | 10 | 6 | 8 |
| **Treated duration** | 2h | 24h | 7h | 4h | 2h | 3h | 5h | 4h | 120h | 8h | 24h |
| **MIDAS** | 99 | 270 | 180 | 115 | 150 | 165 | 180 | 150 | 117 | 115 | 225 |
| **STATUS MIGRAINOSUS & ICTAL MRI DATA** | | | | | | | | | | | |
| **Duration (MRI #1)** | 16d | 87d | 4d | 12d | 9d | 8d | 4d | 7d | 61d | 7d | 4d |
| **Duration (total)** | 42d | 112d | 36d | 25d | 25d | 17d | 9d | 9d | 67d | 13d | 8d |
| **Peak Severity (x/10)** | 10 | 10 | 8 | 8 | 8 | 9 | 8 | 7 | 9 | 8 | 9 |
| **Visual aura*** | N | Y | N | N | N | N | Y* | N | Y | Y* | Y |
| **Non-visual aura*** | N | N | Y* | N | Y* | Y* | Y* | Y* | N | Y* | Y |
| **Time between acute treatment and MRI** | 10h | 12h | 5h | 5h | 10h | 5.5h | 23h | 0h | 0h | 19h | 5h |
| **INTERICTAL MRI-RELATED DATA** | | | | | | | | | | | |
| **Interictal**  **Severity (x/10)** | 2 | 4 | 4 | 3 | 3 | 2 | 0 | 0 | 2 | 0 | 0 |
| *Despite only 3/11 patients having a physician-diagnosis of migraine with aura, during status migrainosus many diagnosed previously as migraine without aura self-reported a visual or non-visual aura as noted here.  ^The month prior to SM, MMD increased to 16.  CM = chronic migraine, d = Days, EM = episodic migraine, F = Female, h = hours, M = Male, MHD = Monthly Headache Days, MIDAS = Migraine Disability Score, MMD = Monthly Migraine Days, N = No, Y = Yes, | | | | | | | | | | | |
